# Trust in federal COVID-19 vaccine oversight and parents’ willingness to vaccinate their children against COVID-19: a cross-sectional study

**DOI:** 10.1186/s12889-024-18342-y

**Published:** 2024-03-16

**Authors:** Hyunmin Yu, José A. Bauermeister, Ufuoma Oyiborhoro, Subhash Aryal, Terri H. Lipman, Andy S. L. Tan, Karen Glanz, Antonia M. Villarruel, Stephen Bonett

**Affiliations:** 1 School of Nursing, University of Pennsylvania, 418 Curie Blvd, Philadelphia, Pennsylvania 19104 USA; 2 Annenberg School for Communication, University of Pennsylvania, 3620 Walnut Street, Philadelphia, Pennsylvania 19104 USA; 3 Perelman School of Medicine, University of Pennsylvania, 3400 Civic Center Blvd, Philadelphia, Pennsylvania 19104 USA

**Keywords:** Vaccine hesitancy, Trust, Federal oversight, Pediatric vaccination, COVID-19 vaccine

## Abstract

**Background:**

Over half of the youth population in the United States, aged 6 months to 17 years, have not received the Coronavirus Disease 2019 (COVID-19) vaccine. Given parents’ central role in vaccinating their children, we examined associations between parents’ trust of the federal oversight of COVID-19 vaccine safety and their willingness to vaccinate their children against COVID-19.

**Methods:**

This cross-sectional study included 975 parents of minor children residing in Philadelphia who completed the online survey between September 2021 and February 2022. Trust was measured using a four-point Likert scale ranging from ‘do not trust’ to ‘fully trust’ for two variables: (1) trust in federal oversight of COVID-19 vaccine safety for children and (2) trust in federal oversight of COVID-19 vaccine safety for the general public. A multiple logistic regression evaluated associations between trust and parents’ willingness to vaccinate their children, which was measured on a five-point Likert scale ranging from ‘strongly disagree’ to ‘strongly agree.’ The analysis was adjusted for race/ethnicity, age, sexual orientation, gender, education, insurance, and parents’ vaccination status.

**Results:**

Analyses included 975 parents whose children had not previously been vaccinated against COVID-19 (mean age 36.79, standard deviation 6.4; 42.1% racial/ethnic minorities; 93.2% heterosexual; and 73.7% with a college degree). Greater trust regarding federal oversight of COVID-19 vaccine safety for children [adjusted odds ratio (aOR) = 1.52, 95% confidence interval (CI): 1.13–2.04] and for the public (aOR = 1.58, 95% CI: 1.17–2.14) were each associated with increased willingness to have their child vaccinated against COVID-19. Unvaccinated parents had decreased willingness compared to parents who had received at least one dose of the vaccine (aOR = 0.14, 95% CI: 0.04–0.41). College-graduate parents exhibited increased willingness compared to those without a college degree (aOR = 2.07, 95% CI: 1.52–2.81). Non-heterosexual parents showed increased willingness compared to heterosexual parents (aOR = 2.30, 95% CI: 1.20–4.76).

**Conclusions:**

Trust in federal COVID-19 vaccine oversight was associated with parental willingness to vaccinate their children against COVID-19 among parents whose children have not yet been vaccinated. Identifying and addressing causes of mistrust are crucial next steps to promote child vaccination. Intervention efforts to address trust gaps should remain a public health priority.

## Background

The Coronavirus Disease 2019 (COVID-19) pandemic, caused by the novel coronavirus SARS-CoV-2, presented an unprecedented global health challenge, necessitating swift and extensive vaccination efforts to curtail the spread of the virus and mitigate its impact on public health [1, 2]. The development of COVID-19 vaccines occurred rapidly due to global collaboration, existing research, and accelerated approval processes. mRNA vaccines emerged quickly, alongside others utilizing established technologies such as viral vectors [3, 4]. The development and distribution of COVID-19 vaccines have been instrumental in the fight against the pandemic, offering a promising avenue for controlling transmission and reducing the severity of the disease [5, 6].

However, the success of vaccine campaigns relies not only on the availability of vaccines but also on the public’s acceptance and willingness to be vaccinated [7]. Vaccine hesitancy, characterized by delayed acceptance or refusal of vaccines despite their availability, remains a significant concern that can impede vaccination efforts [8–10]. Vaccine hesitancy encompasses a spectrum of attitudes and beliefs, including concerns about vaccine safety and effectiveness, mistrust in vaccine manufacturers, and doubts about the regulatory processes governing vaccine approval [11–13].

Understanding vaccine hesitancy, particularly regarding child vaccination, is crucial. As of June 2023, only 36.8% of children under 17 in the United States have received the COVID-19 vaccine. Vaccination rates vary across age groups: 10% for children aged 6 months to 4 years, 32.9% for 5–11 year-olds, 54.9% for 12–15 year-olds, and 69.7% for 16–17 year-olds [14]. It is essential to comprehend factors influencing parental willingness to vaccinate their children against COVID-19 to ensure widespread acceptance and optimal protection.

Trust in federal oversight of vaccine safety, the level of confidence or belief that parents have in the monitoring and regulation conducted by the federal government to ensure the safety of vaccines for children [15–17], is a critical factor contributing to parental vaccine hesitancy, including harboring doubts about the regulatory processes [11–13]. Federal vaccine trust encompasses parents’ faith in the thoroughness, accuracy, and transparency of the oversight processes implemented by federal authorities to guarantee the well-being of individuals receiving the vaccine, particularly among the pediatric population [15–17].

Recognizing the significant impact of historical racism and biased policies in the United States is essential, especially concerning vaccination attitudes. This history has led to widespread mistrust in government and healthcare systems, particularly among certain communities [18]. Rooted in discriminatory practices, such as the Tuskegee Syphilis Study, where African American participants were untreated without consent [19], this mistrust persists due to ongoing healthcare disparities along racial lines [20, 21]. These historical injustices and disparities profoundly influence contemporary issues, including challenges posed by the COVID-19 pandemic. Understanding this context is vital for examining attitudes toward federal oversight of COVID-19 vaccine safety.

A lack of trust can manifest as doubts about the transparency and rigor of vaccine testing, as well as concerns about the impartiality of government agencies overseeing vaccine development and distribution [15–17]. A 2020 meta-analysis on the acceptability of COVID-19 vaccination and its predictors revealed that, in 20 surveyed countries, trust in the government was a significant predictor of willingness to vaccinate [22]. Previous studies predominantly focused on assessing adults’ willingness to be vaccinated themselves, rather than parents’ willingness to vaccinate their children [23–25]. However, it is crucial to investigate whether trust is associated not only with adults’ willingness to vaccinate themselves but also with their willingness to vaccinate their children. Additionally, research on parents’ willingness to vaccinate their children has mainly centered on identifying predictors, rather than examining the hypothesized relationship between trust and parental willingness while also accounting for factors known to be associated with parental willingness [26–28].

This cross-sectional study aimed to investigate the association between parental trust in federal oversight regarding vaccine safety and their willingness to vaccinate their children against COVID-19. Specifically, it examined whether an elevated level of trust was linked to an increased likelihood of willingness to vaccinate after adjusting for factors known to be associated with parental willingness, particularly among parents who had not previously vaccinated their children. Building upon prior research focused on vaccine acceptance and hesitancy [29], we hypothesized that the influence of trust in governmental authorities would also affect the willingness of parents to vaccinate their children. Additionally, we examined various sociodemographic factors that have revealed differences in individuals’ willingness to vaccinate against COVID-19 in prior studies, including gender, age, educational level [22, 28], parental vaccination history [30], race and ethnicity [27, 31], insurance status [32], and sexual orientation [33].

## Methods

### Procedure

This cross-sectional study was conducted as part of the Philadelphia CEAL (Community Engagement Alliance) initiative [34]. The overarching objective of this initiative was to redress disparities evident in COVID-19 testing, vaccine adoption, and participation in clinical trials, particularly within the communities of Philadelphia that experienced a disproportionate impact during the pandemic.

Utilizing a non-probability sampling approach, our online survey was completed by 8,153 participants between September 2021 and February 2022. To ensure data quality, a multi-layered fraud detection system was employed, resulting in the reduction of the final valid sample to 2,870 participants [35]. For the present study, we initially included the subset of 1,309 parent participants, each of whom had at least one child ≤ 17. As our primary focus was on assessing the willingness of parents who had not yet engaged in the behavior, we excluded 296 participants who reported having already vaccinated their children. Additionally, as our objective is to perform analyses within a timeframe unaffected by a significant policy change for a specific age group, we opted to exclude 21 participants who completed the survey before November 2nd, 2021. This date marks the release of guidelines by the Centers for Disease Control and Prevention (CDC) on pediatric COVID-19 vaccination, specifically tailored for the age group of 5–11 years. To enhance the robustness of our analysis and address the limitations posed by the limited sample size, we treated 17 additional participants who chose ‘prefer not to answer’ for sociodemographic variables as having missing data. Therefore, the remaining 975 parent participants were included for analysis. All study procedures received approval from the University of Pennsylvania Institutional Review Board. Written informed consent was obtained from all participants after fully explaining the nature and potential consequences of the study.

### Measures

#### Predictor variable

The primary predictor in this analysis was COVID-19 vaccine trust that was assessed using two questions. First, trust in federal oversight of COVID-19 vaccine safety for public was measured by the question “How much do you trust the federal government to ensure the COVID-19 vaccine is safe for the public?” Second, trust in federal oversight of COVID-19 vaccine safety for children was measured by the question “How much do you trust the federal government to ensure a COVID-19 vaccine is safe for children?” These questions were part of a set of harmonized metrics developed through an iterative process involving CEAL teams across the country and collaborators from the National Institutes of Health (NIH) [36]. Responses were recorded on a four-point Likert scale, with options such as ‘1 = fully trust,’ ‘2 = mostly trust,’ ‘3 = somewhat trust,’ and ‘4 = do not trust.’ These were then reverse-coded. Subsequently, both variables were converted into binary form, combining responses 1 and 2 to indicate minimal or no trust, and categorizing responses 3 and 4 as trust. A Pearson’s correlation was conducted to assess the potential for high inter-item correlation between these two conceptually related variables. The correlation coefficient obtained was 0.35, which falls below the conventional threshold of 0.5 for high correlations [37].

Additionally, we computed generalized variance inflation factors for each independent variable to assess multicollinearity. All variables demonstrated low generalized variance inflation factor values, thus justifying the inclusion of both trust variables in the model.

#### Outcome variable

The outcome measured in this study was parents’ willingness to have their child vaccinated against COVID-19. To assess the outcome variable, a single item was employed: “I am willing to have my child receive a COVID-19 vaccine.” Parents provided their responses on a scale that ranged from ‘1 = strongly disagree’ to ‘5 = strongly agree.’ Additional response options included ‘2 = somewhat disagree,’ ‘3 = neither agree nor disagree,’ and ‘4 = somewhat agree.’ These responses were condensed into a dichotomous format, with ‘0 = disagree/neutral’ representing the merging of responses ‘1,’ ‘2,’ and ‘3,’ and ‘1 = agree’ representing the merging of responses ‘4’ and ‘5.’

#### Covariates

We included various sociodemographic factors that were found to be associated with individuals’ willingness to vaccinate against COVID-19 in previous studies as covariates [22, 25–28, 30–33]. Parents’ sociodemographic characteristics, including race and ethnicity, age, sexual orientation, gender, education attainment, and insurance status, were included as covariates. Additionally, the COVID-19 vaccination status among parents themselves was included as a binary covariate. Due to exceedingly small sample sizes, responses of ‘prefer not to answer’ for gender, sexual orientation, and parental vaccination status were excluded from the regression analysis.

### Statistical analysis

We conducted statistical analysis using R version 4.2.3. Employing multiple logistic regression analysis, we assessed the relationship between parents’ trust in federal oversight of COVID-19 vaccine safety and their willingness to have their child vaccinated against COVID-19, controlling for covariates. To gauge multicollinearity, we calculated generalized variance inflation factors for each independent variable. We computed statistical significance levels using two-sided tests at the alpha level of 0.05, as well as the adjusted odds ratios (aOR) and corresponding confidence intervals (CI).

## Results

The sociodemographic characteristics of the final sample, which comprises 975 parent participants is presented in Table 1. Their average age was 36.79 years, with a standard deviation (SD) of 6.4. Among the parent participants, 57.9% identified as non-Hispanic White, 93.2% identified as heterosexual, and 97.5% of parents had received at least one dose of the vaccine. The majority of parents (65.8%) expressed a willingness to vaccinate their children against COVID-19, while 34.2% reported being unwilling. Additionally, 68.2% of parents expressed trust in federal oversight of COVID-19 vaccine safety for the general public, and 64.1% expressed trust in federal oversight of COVID-19 vaccine safety for children.

**Table 1:** Demographic characteristics

	Parent participants (*n* = 975)
**Willingness (%)**	
Willing	642 (65.8)
Not willing	333 (34.2)
**Trust in federal oversight of COVID-19 vaccine safety for children (%)**	
Trust	625 (64.1)
Not trust	350 (35.9)
**Trust in federal oversight of COVID-19 vaccine safety for the public (%)**	
Trust	665 (68.2)
Not trust	310 (31.8)
**Age (mean (SD))**	36.79 (6.4)
**Race and ethnicity (%)**	
NH-White	565 (57.9)
NH-Multiracial/Other	22 (2.3)
NH-Asian	38 (3.9)
NH-Black or African American	273 (28.0)
Hispanic/Latinx	77 (7.9)
**Gender (%)**	
Woman	635 (65.1)
Man	336 (34.5)
Transgender or gender diverse	4 (0.4)
**Sexual orientation (%)**	
Straight/Heterosexual	909 (93.2)
Non-heterosexual	66 (6.8)
**Education (%)**	
College degree	719 (73.7)
Non-college degree	256 (26.3)
**Insurance (%)**	
Yes	944 (96.8)
No/Don’t know	31 (3.2)
**Vaccination status (%)**	
Yes, at least one dose	951 (97.5)
No, have not gotten any vaccine	24 (2.5)

The adjusted odds ratios of all predictor variables and covariates are presented in Table 2. Greater trust regarding federal oversight of COVID-19 vaccine safety for children was associated with increased willingness among parents to have their child vaccinated against COVID-19 (aOR = 1.52, 95% CI: 1.13–2.04). Greater trust regarding federal oversight of COVID-19 vaccine safety for the public had similar increased willingness (aOR = 1.58, 95% CI: 1.17–2.14). Parents who had not received any COVID-19 vaccines demonstrated a reduced willingness to have their child vaccinated against COVID-19 compared to parents who had received at least one dose of the vaccine (aOR = 0.14, 95% CI: 0.04–0.41). Parents with a college degree exhibited an increased willingness for pediatric vaccination compared to those without a college degree (aOR = 2.07, 95% CI: 1.52–2.81). Non-heterosexual parents showed a higher degree of willingness compared to heterosexual parents (aOR = 2.30, 95% CI: 1.20–4.76). There were no significant associations found between insurance status, parental age, gender, race, and ethnicity with parental willingness to vaccinate their children.

**Table 2:** Multivariate analysis of variables that predicted willingness to have child vaccinated (*n* = 975)

Predictor variables and covariates	Adjusted odd ratios (95% confidence interval)	p-value
**Trust in federal oversight of COVID-19 vaccine safety for children**	1.52 (1.13–2.04)	0.005*
**Trust in federal oversight of COVID-19 vaccine safety for the public**	1.58 (1.17–2.14)	0.003*
**Parental vaccination status**		
Received at least one dose (reference)		
Have not gotten any vaccine	0.14 (0.04–0.41)	0.001*
**Age**	1.02 (0.99–1.05)	0.055
**Race and ethnicity**		
NH-White (reference)		
NH-Multiracial/Other	1.51 (0.55–4.59)	0.438
NH-Asian	0.89 (0.44–1.87)	0.751
NH-Black or African American	0.95 (0.69–1.31)	0.763
Hispanic/Latinx	1.28 (0.74–2.30)	0.383
**Gender**		
Woman (reference)		
Man	1.09 (0.82–1.47)	0.551
Transgender or gender diverse	0.49 (0.05–5.79)	0.547
**Sexual orientation**		
Straight or heterosexual (reference)		
Non-heterosexual	2.30 (1.20–4.76)	0.017*
**Education**		
Non-college degree (reference)		
College degree	2.07 (1.52–2.81)	< 0.001*
**Insurance**		
No/Don’t know (reference)		
Yes	1.23 (0.52–2.78)	0.626

^*^statistical significance at the level of 0.05

## Discussion

This study described parental trust in federal oversight of COVID-19 vaccine safety and its impact on the willingness to vaccinate children against COVID-19. As of June 2023, 63.2% of the youth population, spanning ages 6 months to 17 years, remains unvaccinated [14]. This statistic underscores the timeliness and relevance of our investigation, conducted during the data collection period from September 2021 to February 2022. Trust in the federal oversight of COVID-19 vaccine safety, both for the general public and specifically for children, is associated with parental willingness to vaccinate their children against COVID-19. The parallel nature of these findings suggests that cultivating trust in regulatory processes for both children’s and public vaccine safety could have a synergistic impact on increasing overall vaccine acceptance. To the best of our knowledge, no prior reports or studies had focused on the association between parental trust in federal COVID-19 vaccine oversight and parental willingness to have their child receive a COVID-19 pediatric vaccination. Mistrust related to COVID-19 has been identified as a significant barrier to higher vaccine acceptance and treatment compliance [38]. Investigating these concerns, including doubts about safety and efficacy, particularly in the context of pediatric COVID-19 vaccination, is crucial because children and adolescents play a pivotal role in achieving herd immunity [39].

The association between parental trust in federal oversight of COVID-19 vaccine safety and an increased pediatric vaccination willingness is consistent with prior research on vaccine hesitancy. Existing studies have underscored the critical role of trust in public health agencies and vaccine regulatory bodies [12, 17, 25, 40]. When parents hold reservations or uncertainties regarding vaccine safety monitoring and regulation, their willingness to vaccinate their children may diminish, driven by concerns about their children’s well-being. This underscores the urgency of establishing transparent and effective communication channels from public health authorities [41]. It is also essential to involve community messengers to address parental apprehensions and foster trust in vaccine safety monitoring systems. However, it is important to acknowledge that we did not investigate whether parental trust of COVID-19 vaccines may be influenced by their experiences with childhood vaccinations. Future research could explore the association between parental perceptions of COVID-19 vaccine safety and their confidence in routine childhood vaccinations. Such insights would contribute to a more comprehensive understanding of vaccine hesitancy dynamics and inform targeted interventions to address concerns and build trust across various vaccination contexts.

The association between parental COVID-19 vaccination status and their willingness to vaccinate their children further underscores the impact of personal vaccination experiences on decision-making. Previous studies have indicated that influenza vaccination history strongly predicts COVID-19 vaccine acceptance [22]. Similarly, parents who have chosen not to receive the COVID-19 vaccine themselves were also more hesitant to vaccinate their children [27]. Interventions aimed at increasing COVID-19 vaccination rates among adults may indirectly contribute to higher pediatric vaccination rates [42]. Moreover, addressing the concerns and barriers faced by unvaccinated parents is vital in promoting comprehensive family vaccination.

Conversely, the findings reveal that parents with higher levels of education were more inclined to vaccinate their children. This outcome aligns with existing research emphasizing the positive association between educational attainment and vaccine acceptance [22, 26, 40, 43]. Educated parents may have better access to credible information and resources, enabling them to make informed decisions about vaccination. They may also have a heightened awareness of the benefits of vaccination and the risks associated with vaccine-preventable diseases. Additionally, parents with higher levels of education are likely to have limited exposure to the untrustworthy histories of federal agencies and healthcare. Public health initiatives should prioritize targeted educational interventions. These initiatives should be designed to provide accessible and credible information to all parents, ensuring they are equipped to make informed decisions regarding vaccination. Collaborations with educational institutions, community organizations, and healthcare providers can enhance the reach and effectiveness of these educational campaigns. Moreover, policymakers should carefully weigh the potential impact of historical mistrust on the implementation and success of public health policies. Emphasizing transparency and accountability becomes crucial in rebuilding trust within communities.

The finding that non-heterosexual parents displayed increased willingness to vaccinate their children aligns with a prior study [33] that reported higher COVID-19 vaccination coverage and vaccine confidence among gay or lesbian adults compared to heterosexual adults. However, it also underscores the critical need for more comprehensive and inclusive research into COVID-19 vaccine hesitancy for lesbian, gay, bisexual, transgender, queer (or questioning), and other sexual and gender diverse individuals (LGBTQ+). The existing systematic reviews reveal substantial gaps in this area, with many studies failing to adequately account for LGBTQ + individuals (often limiting questions to binary gender choices without inquiring about sexual orientation) or gather sufficient data to explore vaccine hesitancy and its underlying factors [44, 45]. Addressing these limitations through more inclusive research is imperative to gain a nuanced understanding of vaccine acceptance within this population and to inform tailored public health strategies that can effectively promote vaccination and mitigate disparities.

The lack of significance for certain variables such as gender, insurance status, race, and age in our study examining parental willingness to vaccinate their children against COVID-19 may be attributed to several factors. First, factors associated with adults’ intentions to receive the COVID-19 vaccine themselves may not necessarily translate to the intentions of parents regarding their children’s vaccination. Parental decision-making involves additional considerations such as perceived risks and benefits specific to their children’s health and well-being, which may differ from individual vaccination decisions [46]. Moreover, it is possible that trust in federal oversight mediates the relationship between these sociodemographic factors and parental willingness to vaccinate, such that sociodemographic differences in trust were driving the associations with willingness seen in other studies. Future research with larger sample sizes and more comprehensive measures could explore these potential causal pathways to better understand the nuanced influences on parental vaccination decisions.

There are several limitations to this study. First, we did not collect information on the ages of the children and their perceived access to vaccination, and as a result, we were unable to account for this potentially important covariate in our analysis. Second, our findings are based on cross-sectional survey data that cannot assess the longitudinal associations between parents’ trust in federal COVID-19 vaccine oversight and their willingness to vaccinate their children. Also, the survey was administered via an online survey platform and online outreach was a primary recruitment strategy. Therefore, study participation was limited to individuals who have access to the internet on web-enabled devices. Additionally, our study was limited to residents of Philadelphia, where a significant proportion of participants were vaccinated, insured, and had higher levels of education. As a result, our findings and conclusions may be limited in their generalizability. Finally, it is essential to note that our study focused on parental willingness to vaccinate their children, which may not always align with actual vaccination behavior. These limitations should be carefully considered when interpreting the results of this study.

The greatest strength of our study is its novel approach in assessing the parents’ trust in association with COVID-19 pediatric vaccination. Although there are many studies on parents’ attitudes toward childhood vaccination, our study primarily examines how trust in the federal oversight of COVID-19 vaccine safety, both for the general public and specifically for children, can affect parents’ willingness to vaccinate their children against COVID-19. This study provides insights into this parental vaccination hesitancy for health communication practitioners, vaccination advocates, and the public health workforce. It also highlights the need for further research and evaluation of parent-focused COVID-related information materials and interventions.

## Conclusions

Trust in federal COVID-19 vaccine oversight, both for the general public and specifically for children, is associated with parental vaccination willingness. Understanding the root causes of mistrust in federal oversight is crucial for tailoring vaccination promotion efforts and addressing hesitancy among specific demographic groups. Further research and targeted interventions should aim to address the underlying reasons behind these associations, ultimately working toward higher pediatric vaccination rates and achieving broader public health goals.

## Data Availability

The datasets used and/or analyzed during the current study are available from the corresponding author on reasonable request.

## Abbreviations

COVID-19: Coronavirus Disease 2019
CDC: Centers for Disease Control and Prevention
SD: Standard Deviation
CEAL: Community Engagement Alliance
aOR: Adjusted Odds Ratio
CI: Confidence Interval
LGBTQ+: Lesbian, Gay, Bisexual, Transgender, Queer (or Questioning), and other sexual and gender diverse individuals
NIH: National Institutes of Health
